# Co-inoculation of a Pea Core-Collection with Diverse Rhizobial Strains Shows Competitiveness for Nodulation and Efficiency of Nitrogen Fixation Are Distinct traits in the Interaction

**DOI:** 10.3389/fpls.2017.02249

**Published:** 2018-01-10

**Authors:** Virginie Bourion, Karine Heulin-Gotty, Véronique Aubert, Pierre Tisseyre, Marianne Chabert-Martinello, Marjorie Pervent, Catherine Delaitre, Denis Vile, Mathieu Siol, Gérard Duc, Brigitte Brunel, Judith Burstin, Marc Lepetit

**Affiliations:** ^1^Agroécologie, INRA, AgroSup Dijon, Université Bourgogne Franche-Comté, Dijon, France; ^2^Laboratoire des Symbioses Tropicales et Méditerranéennes, INRA, IRD, CIRAD, Montpellier SupAgro, Université de Montpellier, Montpellier, France; ^3^Laboratoire d'Ecophysiologie des Plantes Sous Stress Environnementaux, INRA, Montpellier SupAgro, Université de Montpellier, Montpellier, France

**Keywords:** partner choice, competitiveness for nodulation, pea-rhizobium symbiosis, nitrogen fixation efficiency, genetic diversity, plant breeding, *Pisum sativum*, *Rhizobium leguminosarum* sv. *viciae*

## Abstract

Pea forms symbiotic nodules with *Rhizobium leguminosarum* sv. *viciae* (Rlv). In the field, pea roots can be exposed to multiple compatible Rlv strains. Little is known about the mechanisms underlying the competitiveness for nodulation of Rlv strains and the ability of pea to choose between diverse compatible Rlv strains. The variability of pea-Rlv partner choice was investigated by co-inoculation with a mixture of five diverse Rlv strains of a 104-pea collection representative of the variability encountered in the genus *Pisum*. The nitrogen fixation efficiency conferred by each strain was determined in additional mono-inoculation experiments on a subset of 18 pea lines displaying contrasted Rlv choice. Differences in Rlv choice were observed within the pea collection according to their genetic or geographical diversities. The competitiveness for nodulation of a given pea-Rlv association evaluated in the multi-inoculated experiment was poorly correlated with its nitrogen fixation efficiency determined in mono-inoculation. Both plant and bacterial genetic determinants contribute to pea-Rlv partner choice. No evidence was found for co-selection of competitiveness for nodulation and nitrogen fixation efficiency. Plant and inoculant for an improved symbiotic association in the field must be selected not only on nitrogen fixation efficiency but also for competitiveness for nodulation.

## Introduction

Legumes are a sustainable source of protein for both human and animal diets. Owing to their ability to establish symbiosis with nitrogen-fixing bacteria, their cultivation is vital for reducing the use of nitrogen fertilizers, a major cause of agricultural greenhouse gas emissions and energy consumption (Jensen and Hauggaard-Nielsen, 2003; Galloway et al., 2008). The biological nitrogen fixation (BNF) obtained from grain legume crops (pulses and oilseed legumes) represents a quarter of the N applied to arable lands annually as chemical fertilizers (Herridge et al., 2008). Despite these benefits, grain legumes are under-cultivated in European agricultural systems. They suffer from lower productivity and more variable yields than cereals whose production depends on high fertilizer inputs. Improving the regularity of grain legume yield is thus a major objective from an agroecological standpoint, and one avenue to achieve it could be through improved symbiosis.

Pea (*Pisum sativum* L.) is one of the world's most cultivated pulse crops (Duc et al., 2015). It was one of the first domesticated crops and was an important component, along with other legumes and cereals, of the diet of early civilizations in the Middle East and the Mediterranean Basin (Zohary and Hopf, 1973; Cousin, 1997; Smykal et al., 2015). Wild pea relatives include the species *P. fulvum* and *P. sativum* subsp. *elatius*. Cultivated pea appeared in the early Neolithic period in the area of the Fertile Crescent, and later spread throughout Europe, through Turkey, Greece and the Caucasus, and eastwards to India and China through Iran and Afghanistan. Cultivated peas mostly belong to *P. sativum* subsp. *sativum*. *P. sativum* subsp. *abyssinicum* is a less frequently cultivated pea, restricted to Yemen and Ethiopia (Vershinin et al., 2003; Jing et al., 2010; Smykal et al., 2011).

Establishment of the symbiosis involves mutual recognition of the plant legume and rhizobial partners in the soil, followed by the development of root symbiotic organs called nodules in which the bacteria fix dinitrogen. In most cases, the symbiotic interaction between pea and rhizobia involves strains of *Rhizobium leguminosarum* symbiovar *viciae* (Rlv). Rlv has long been regarded as able to nodulate all the species of the legume tribe Viciae. However, differences in symbiotic host range within the Viciae tribe have been reported between Rlv strains (Laguerre et al., 2003; Mutch and Young, 2004). Sequence variation in the Rlv symbiotic *nod* genes involved in Nod factor production has been described (Laguerre et al., 2001; Kumar et al., 2015; Peix et al., 2015). Variation of Rlv host specificity within the genus *Pisum* was one of the earliest reported cases of host-controlled restriction of nodulation. Some cultivated peas from Afghanistan and Middle East were identified as being resistant to nodulation by European Rlv strains, and requiring specific Rlv strains found in Israel, Turkey or Afghanistan to nodulate, whereas European pea cultivars were nodulated by both European and Middle Eastern strains when mono-inoculated (Lie, 1978; Young and Matthews, 1982). The pea *SYM2* and bacterial *nodX* genes confer this specific interaction (Holl, 1975; Davis et al., 1988). Such variations in both bacterial *nod* genes and legume genes encoding receptor-like kinases (LysM-RLKs) required for Nod factor perception indicate them to be major determinants of the specificity of a legume-rhizobia association (Spaink et al., 1991; Denarie et al., 1992; Walker et al., 2000; D'Haeze and Holsters, 2002; Liang et al., 2014).

In the complex soil environment, legume roots are exposed to heterogeneous rhizobial populations containing multiple compatible strains (Laguerre et al., 2003; Mutch and Young, 2004; Bourion et al., 2007; Sachs et al., 2009). Various data indicate that BNF could be suboptimal in pea as natural Rlv populations are quantitatively and qualitatively heterogeneous, which often results in nodulation of peas by poorly efficient rhizobia (Fesenko et al., 1995; Laguerre et al., 2007). There is general agreement about the interest of rhizobial inoculation of pea with selected Rlv bacteria for improving BNF (Bremer et al., 1988; Fesenko et al., 1995; McKenzie et al., 2001). However, even when pea seeds are inoculated with efficient Rlv strains, these may be outcompeted by naturally occurring rhizobia (Meade et al., 1985). Partner choice is not only determined by the abundance of the various bacteria but also depends on the competitiveness for nodulation of these strains (Triplett and Sadowsky, 1992; Laguerre et al., 2003). Therefore, understanding mechanisms behind competitiveness for nodulation in pea may lead to improved inoculation strategies.

The nodulation process has a high metabolic cost for both rhizobial and legume partners (Phillips, 1980; Schulze et al., 1999; Trainer and Charles, 2006; Voisin et al., 2007). The establishment of the symbiotic interaction is also a complex evolutionary process where the interests of the host and the bacteria are not always aligned. There seem to be mechanisms allowing the plant to monitor the nitrogen-fixation performance of symbiotic bacteria and to sanction the inefficient strains (Simms and Taylor, 2002; Oono et al., 2011). However, poorly-fixing rhizobial strains often gain advantage over beneficial strains despite offering poor growth benefit to their plant host, indicating that additional factors may influence fitness of symbiotic bacteria (Marco et al., 2009; Sachs et al., 2010; Fujita et al., 2014). Domesticated pea and faba bean crops tend to have fewer compatible symbionts than their wild relatives (Mutch and Young, 2004). Whether modern cultivars are less able to establish beneficial associations than older landraces in these species, as was observed for soybean, remains to be seen (Kiers et al., 2007).

The genetic basis of pea-Rlv partner choice when roots are exposed to a mixture of compatible Rlv strains is far from being fully understood. This study investigates the genetic variability of partner choice in the pea-Rlv symbiosis. A pea core-collection representative of the genetic and biogeographic diversity found within the genus *Pisum* was inoculated with a mixture of five diverse compatible Rlv strains. Differences in Rlv choice according to pea diversity and selection history were investigated. The relationship between nodulation competitiveness and nitrogen fixation efficiency was evaluated. Consequences for breeding and inoculation strategies for improving symbiotic traits in the pea crop are discussed.

## Materials and methods

### Biological material

One hundred and four *Pisum* accessions were selected from the reference pea collection available at INRA Dijon (http://www.thelegumeportal.net/), according to their diversities of genetic or geographical origin and their variability in agronomic traits, such as their cultivation status, end-use and sowing type (Burstin et al., 2015). This 104-pea collection includes 12 wild or semi-wild genotypes, 36 landraces, 12 inbred lines or germplasm, and 44 cultivars (Table S1). Accessions originate from as many as 36 countries, from known centers of diversity and domestication (Middle East, Ethiopia, Afghanistan) or from areas where domesticated peas were subsequently disseminated, in Southern Africa, Asia, Europe and America. Among the wild, semi-wild or landrace genotypes, two are *P. fulvum* accessions and nine are *P. sativum* accessions identified as belonging to the subspecies *abyssinicum, elatius* or *humile*. The panel of 44 cultivars is representative of the variation in end-use, sowing type and other characteristics of peas cultivated since the end of seventeenth century.

The five selected Rlv strains were previously identified as nodulating pea (Table S2) and have diverse geographical origins. SA (P1NP2H) and SD (P1NP2K) are two strains originating from France (Laguerre et al., 2003). SE (SL16) is a strain collected in Algeria. SK (SpR) and SF (RifR) are spontaneous antibiotic mutants, respectively of the reference strain 3841 originating from England (Brewin et al., 1980, 1983), and of the strain TOM originating from Turkey and known to be required for nodulation by some Afghan peas (Winarno and Lie, 1979; Young et al., 1982).

### Pea collection genetic structure analyses

The 104 pea accessions were genotyped using the GenoPea 13.2 K SNP Array (Tayeh et al., 2015). A filtering was performed to exclude highly heterozygous SNPs, which are not expected given the high selfing rate in pea, and SNPs with a minor allele frequency of <0.02. Following these steps, a set of 11,218 markers was used for population structure analyses. The genetic structure of the sample was investigated using two methods: (1) a model-based Bayesian clustering assignment algorithm implemented in the software fastSTRUCTURE (Raj et al., 2014) and (2) a discriminant analysis of principal components—DAPC—a multivariate method which employs PCA to reduce the number of correlated variables (SNP markers) to be analyzed using a discriminant analysis implemented in the R package Adegenet (Jombart et al., 2010). The fastSTRUCTURE analysis was run for a number of clusters (K) ranging from 1 to 20 with 5 replicates per *K*-value and using the “simple prior” option (Figure S1). To evaluate the repeatability of runs and check for the absence of true multimodality, the program CLUMPP v.1.1.2 was run using the Greedy algorithm (Jakobsson and Rosenberg, 2007). The putative optimal number of clusters was assessed from the likelihood profile and admixture plots were obtained using a custom python script. The second method, DAPC, was run without prior knowledge of groups. The optimal number of clusters was thus assessed through sequential K-means and model selection using the Bayesian information criterion. The number of principal components was determined to be 2 through maximization of the α-score measuring the difference between the proportion of successful reassignment of the analysis and values obtained using random groups.

### Growth conditions and phenotyping

Plants were grown in a greenhouse under controlled temperature (21/16°C) in a 16/8-h day-night cycle and under a mean photosynthetically active radiation of 250 μmol photons m^−2^ s^−1^ furnished with high-pressure sodium lamps. Prior to sowing in pots, the seeds of *P. fulvum* and of some *P. sativum* subsp. *elatius* or *humile* accessions were scarified, and the seeds of all the accessions sterilized in 10% bleach for 10 min and rinsed five times in water. Seeds were sown on square 2-L pots previously sterilized and filled with a 1:1 (v/v) mixture of previously sterilized attapulgite and clay balls (2–6 mm diameter). Pots were top-watered from day one until 8 days after sowing.

In a first experiment (E1), a three-block randomized design was used, with two pots per accession in each block and three seeds sown per pot. At sowing, each seed was inoculated with 1 mL (~10^8^ cfu) of a rhizobial inoculum comprising an equal proportion of the five Rlv strains. Eight days after sowing, two of the three plantlets were kept per pot, always removing, when possible (i.e., when three plants were present), the plant from the same corner irrespective of its growth characteristics. To allow both nodulation and sufficient plant growth to take place (Moreau et al., 2008; Bourion et al., 2010), they were supplied with a low nitrate content (0.625 mM) nutrient solution (Table S3). Four weeks after sowing, all 1,248 plants kept were harvested. For each accession, the nodules formed on roots were counted on one of the two plants in one pot per block and their total dry matter determined. Rhizobia were isolated for each accession from a sample of 60 nodules randomly collected on the two plants of the other pot of the block. After nodule surface sterilization, each nodule was crushed and undifferentiated bacteria cultivated on YM agar plates. Bacterial identities of individual nodules were determined either by assaying antibiotic resistance on YM medium supplemented with Rif (300 mg L^−1^) for SF strain and with Sp and St (each at 300 mg L^−1^) for SK strain and by PCR amplification using specific primers for SA, SD and SE (Table S2). In most cases the bacteria present in each nodule were clonal and we found little evidence for multiple infection (mixed nodule) (<1%; data not shown). The frequency of nodules containing each strain was then calculated for each pea genotype, allowing estimation of the competitiveness of the strains.

In a second experiment (E2), 18 pea accessions selected from the 104 were mono-inoculated with each of the five Rlv strains or, as a control, without any inoculation (NI). Each mono-inoculation (or lack of inoculation) was performed in one assigned bank not adjacent to the others, in order to prevent cross-contamination. A four-block randomized design was used, with one pot per accession in each block, and four seeds sown per pot. At sowing, except for the NI control, each seed was inoculated with a 1 mL cell suspension of one of the five Rlv strains (~10^7^ cfu). Eight days after sowing, two of the four plantlets were harvested in each pot, always when possible from the same two diagonally opposing corners. The absence of nodules was confirmed in the plants from the NI bank. The 842 remaining plants were supplied with a nutrient solution without any N until harvest time, 5 weeks after sowing (Table S3). For each pea accession inoculated, all the nodules formed on the two plants were counted, and a sample of 16 nodules was collected over the four blocks. Nodule identity was checked using the same method as in E1. The few cross-contaminated plants were discarded and the shoot dry matter of the remaining plants determined. Under mineral N-free conditions, symbiotic N acquisition is generally the main limiting factor for plant growth and the shoot dry matter determined for each legume-Rhizobium association is indicative of its nitrogen fixation efficiency, as illustrated in both the model legume *Medicago truncatula* and pea (Laguerre et al., 2007, 2012; Moreau et al., 2008; Voisin et al., 2010). In order to compare the efficiency of N fixation while controlling for differences in growth potential between pea accessions, we calculated a normalized shoot dry matter index. For a given strain and pea accession, the index was calculated by dividing the shoot dry matter obtained by the mean shoot dry matter of this pea accession mono-inoculated with all five strains (Heath and Tiffin, 2009). Likewise, a nodulation index was calculated, as the nodule number of a pea accession inoculated with a strain divided by the mean nodule number of this pea accession.

## Results

### Genetic diversity of the pea collection and of the Rlv strains

**Figure 1:**
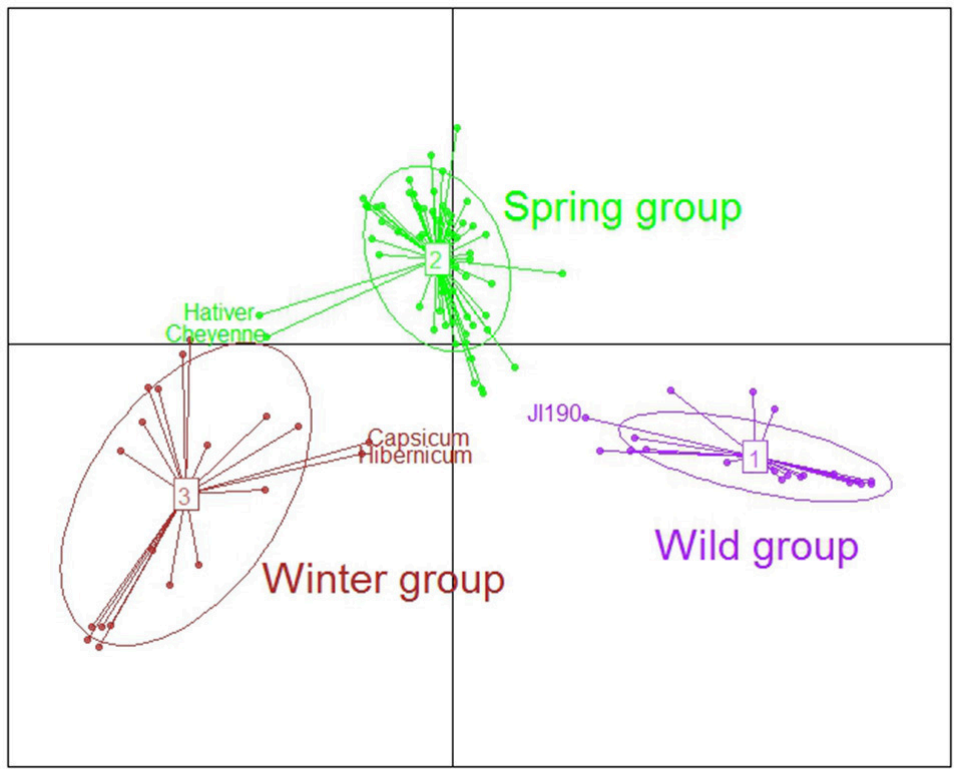
Genetic structure of the 104 accessions of the pea collection using discriminant analysis on principal components (DAPC). After filtering, a set of 11,218 SNPs was used for these analyses (see main text). The first discriminant axis segregated wild vs. cultivated spring or winter peas. The wild group comprises wild or semi-wild accessions and landraces from centers of domestication. The second axis mainly discriminates spring and winter cultivated peas. Note the ambiguous positions of three landraces or traditional cultivars, “JI190” originating from Sudan, “Pisum sativum-Hibernicum JI1846” from Egypt and “Capsicum” from Azerbaidjan and of two winter European cultivars “Hativer” and “Cheyenne”.

Two complementary methods, DAPC and fastSTRUCTURE, were used to assess the population genetic structure of the 104 accessions of the pea collection. After filtering, a set of 11,218 SNPs among the 13,204 of the GenoPea SNP Array (Tayeh et al., 2015) were used for these analyses. The DAPC analysis uncovered three genetic groups and all accessions except five had a membership probability to one of them higher than 0.95 (Table S1, Figure 1). There was a clear distinction between the three groups according to the cultivation status and type of sowing of the accessions. D1 was named “Wild group” as it comprised nine of the 12 wild or semi-wild accessions plus 12 *Pisum sativum* landraces from Abyssinia, Afghanistan and Asia (Table S1). Among these landraces, “JI190,” originating from Sudan, had a membership probability to D1 lower than 0.70. The D2 “Spring group” comprised 64 accessions of which 48 of the 51 accessions were spring sowing types. The D3 “Winter group” comprised 18 genotypes among which 15 were winter and three were spring sowing types. Two of these accessions identified as spring sowing types were sampled at an altitude of more than 2000 meters (JI1844 and JI1431; JIC Pisum Collection database, https://www.seedstor.ac.uk), supporting the cold tolerance characteristics of D3. “Pisum sativum-Hibernicum JI1846” identified in the JIC database as a spring sowing type was less related to the D3 group. Three other accessions, “Capsicum” of unknown sowing type, and the two winter cultivars “Hativer” and “Cheyenne” also displayed an ambiguous position between D2 and D3.

The fastSTRUCTURE analysis identified 10 different clusters (K01–K10) which were interestingly found to further subdivide the three DAPC groups (Figure S2). Based on the study of the 70 accessions with cluster membership probabilities higher than 80%, the cluster assignments were found to correlate with species affiliation or geographic origin and breeding history (Table S1). Of the three clusters subdividing the “Wild D1” group, K01 grouped the two *P. sativum* subsp. *abyssinicum* and of wild accessions, the two *P. fulvum* and three *P. sativum* subsp. *elatius* or *humile*, originating from the Middle East. K02 included three of the four accessions from Afghanistan, a *P. sativum* subsp*. humile* and all the three accessions from Nepal or India. K03 consisted of three *P. sativum* from Abyssinia (Ethiopia, Sudan) or Libya. Within the D2 “Spring group,” K04, mainly consists of landraces or fodder peas from Baltic States, Ukraine, Russia or Sweden (“Torsdag,” registered in 1925). Garden peas have been separately selected in France, Great Britain and the Netherlands from the eighteenth century until the end of the 1960's, which is consistent with their separation into the three distinct clusters: K05, with old French garden pea cultivars (including “Corne de Bélier,” 1818); K06 with English garden peas (including “Téléphone à rames,” 1878); and K07, with two Dutch accessions and a *P. sativum* subsp. *elatius* (JI1703) whose origin is unknown. K08, the fifth cluster in D2, grouped French spring dry pea cultivars (including “Baccara,” the most cultivated during the 1990s). It is representative of the change in end-use, from garden toward dry field peas for animal feed, which occurred in Europe from the 1970's. Within the D3 group, K09 and K10 gathered respectively the winter European fodder peas and the French winter pea cultivars (including “Frisson,” 1979).

Phylogenetic analyses based on symbiotic *nod* markers have shown that the five strains used in this study (SA, SD, SE, SK, and SF) are representative of a large diversity within the symbiovar *viciae* (Figure S3).

### Natural variability in pea nodulation and pea-Rlv partner choice

**Figure 2:**
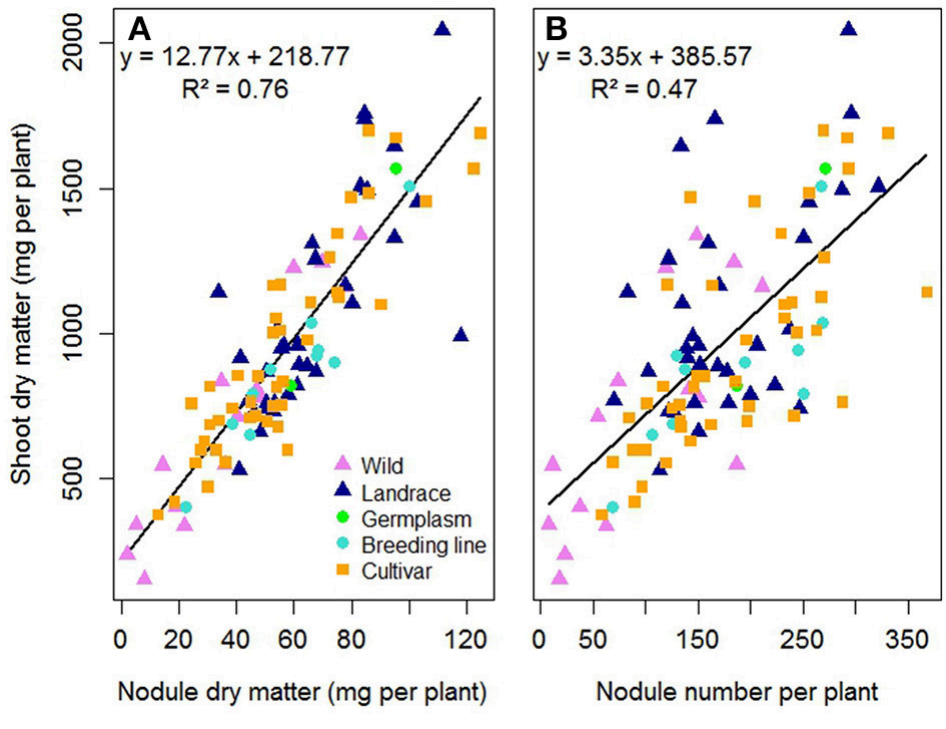
Relationship between shoot dry matter and **(A)** nodule dry matter or **(B)** nodule number per plant, for 104 pea accessions multi-inoculated with a mixture of five Rlv strains (E1 experiment). Each point represents mean values for one pea accession measured 4 weeks after sowing. Symbols are different and colored according to cultivation status. Lines and equations represent linear regression results (both *P* < 0.001).

In the first experiment E1, the 104 pea accessions were assessed for their ability to form nodules and for their choice among the five Rlv strains. Significant variation in nodule biomass and number were observed among the 104 pea accessions when co-inoculated with an equal mixture of the five Rlv strains (Table S4). The lowest biomasses were observed in the wild accessions in K01 (*P. fulvum, P. sativum* subsp. *elatius* or *humile*) or in the fodder and dry pea cultivars belonging to K08, K09, and K10, whereas the highest ones were obtained by landraces or garden pea cultivars (K04, K05, or K07; Figure S4). A significant positive correlation was observed between shoot dry matter and both nodule dry matter and number (*r*^2^ = 0.76, *p* < 0.0001 and *r*^2^ = 0.47, *p* < 0.0001 respectively). No significant effect of the status of the pea accessions (wild, landrace, germplasm, breeding line or cultivar) was observed in these relationships (Figure 2, Table S5).

**Figure 3:**
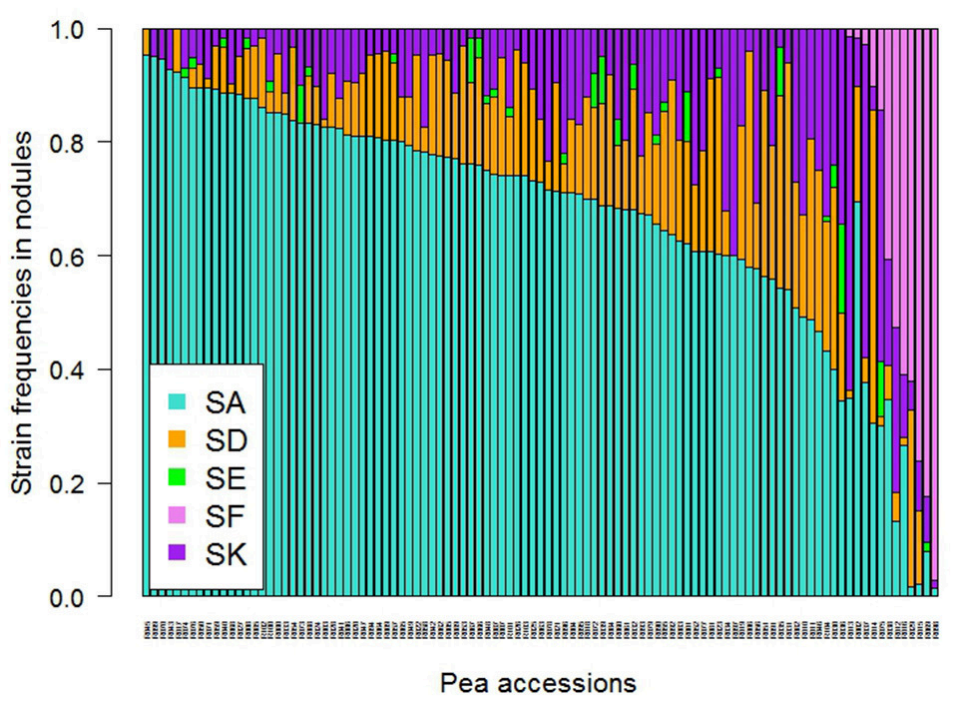
Strain frequencies in the nodules of 104 pea accessions multi-inoculated with a mixture of five Rlv strains (E1 experiment). Rhizobia were isolated for each pea accession from a sample of 60 nodules randomly collected on two plants 4 weeks after sowing.

**Figure 4:**
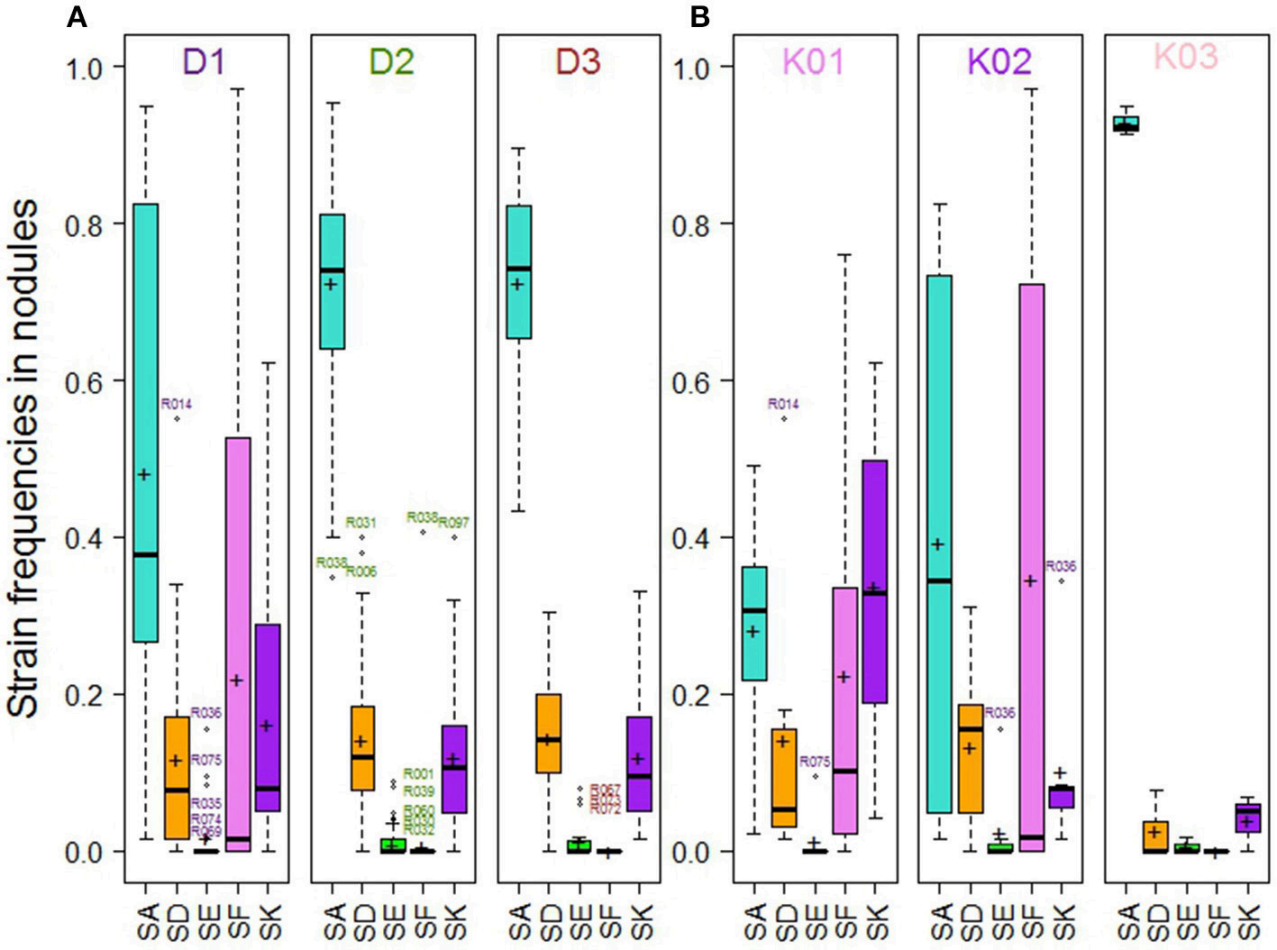
Strain frequencies in the nodules of pea accessions multi-inoculated with a mixture of five Rlv strains (E1 experiment), **(A)** within the 104 pea accessions according to their membership to DAPC groups, **(B)** within the subset of pea accessions belonging to D1 according to their membership to K01, K02 or K03 clusters. Crosses and bold lines respectively indicate mean frequency values and medians. Open circles represent outliers identified by accession numbers.

A large variability in the relative frequency of nodule formation by the five Rlv strains was observed among the 104 pea accessions (Figure 3). With a mean frequency value of 67% among the 104 accessions, globally SA was the most competitive strain, far ahead of SD (14%), SK (13%), SF (5%), and SE (2%) (Figure S5). However, variation between pea accessions was found around these mean values, and differences were observed according to their membership to DAPC groups (Figure 4A, Table S6). The “Wild group” D1 presented a higher diversity in Rlv choice than the groups D2 and D3 which included all the pea cultivars. The relative frequency of SA varied much more in D1 than in D2 or D3 in which it was always higher than 40%. SF was detected in members of D1 with a frequency up to 97% and in none of the members of D2 or D3 except R038 (“Capsicum”). The maximum of the SK relative frequency was higher in D1 than in D2 or D3. Differences were also observed between the three clusters in the D1 group (Figure 4B, Table S6). In K01, very few accessions had a strain preference (i.e., frequency higher than 80%). In K02, the relative frequencies of SA and SF were highly variable, with a strain preference either for SA or SF. In K03 all the accessions had a relative frequency of SA higher than 91% and none was nodulated by SF.

### Correlation between competitiveness and efficiency

To investigate whether competitiveness for nodulation was related with nitrogen fixation efficiency, 18 pea accessions were inoculated separately with each of the five Rlv strains, and their shoot biomasses measured in absence of mineral N supply (E2 experiment). The 18 accessions were selected from the E1 experiment as displaying contrasted partner choice (Figure S6A). They belonged either to the D1 or the D2 group and were representative of the shoot and nodule biomass variations encountered within the pea collection (Figure S6B, Table S1). In almost all pea-Rlv combinations tested we could observe nodulation, indicating that all five strains were able to form nodules regardless of the plant genotype even though the number of nodules varied drastically (Figure S7). The mean values of shoot dry matter obtained for the five strains were correlated to mean nodule numbers (*r*^2^ = 0.62, *p* < 0.0001; Figure S8A). Both pea genotype and Rlv strain were found to have significant effects on nodulation and shoot dry matter of the 90 pea-Rlv combinations, and the interaction between the two factors was significant for both traits (Table S7, Figures S7, S9). Mean values of shoot dry matter obtained for the 18 accessions over the five strains were significantly and positively correlated with those obtained in E1 (*r*^2^ = 0.51, *p* < 0.001; Figure S8B), probably because a large part of the variation is related to differences in growth potential among the pea accessions. To overcome this problem of growth potential differences, we calculated a normalized index for both shoot dry matter and nodulation (see Mat and Meth).

**Figure 5:**
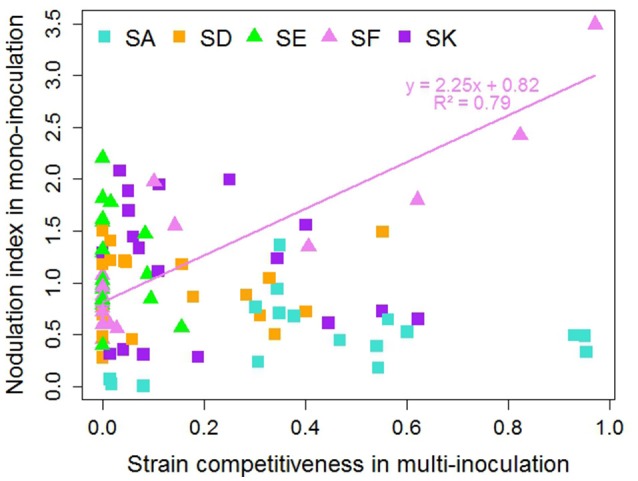
Relationship between nodulation index in mono-inoculation (E2 experiment) and strain competitiveness for nodulation in multi-inoculation (E1 experiment) for 18 contrasted pea accessions. Squares represent European strains. Line and equation represent significant linear regression results (*P* < 0.001).

The nodulation indexes obtained with SA, SD, SE, and SK were not correlated to the competitiveness for nodulation of these strains, as evaluated in E1 (Figure 5, Table S8). As such, the SE strain displayed an overall high ability to form nodules but in all cases had low competitiveness, whereas SA was the most competitive strain in multi-inoculation despite its low nodulation ability. SF was the only strain for which a correlation between the nodulation index and its competitiveness could be detected (*r*^2^ = 0.79, *p* < 0.000001). Indeed, SF was the most competitive with three accessions belonging to K02 (R098 and R029 from Afghanistan; R069 from Israel) having the highest nodulation index, and not competitive at all with the numerous accessions having a low nodulation index with it.

**Figure 6:**
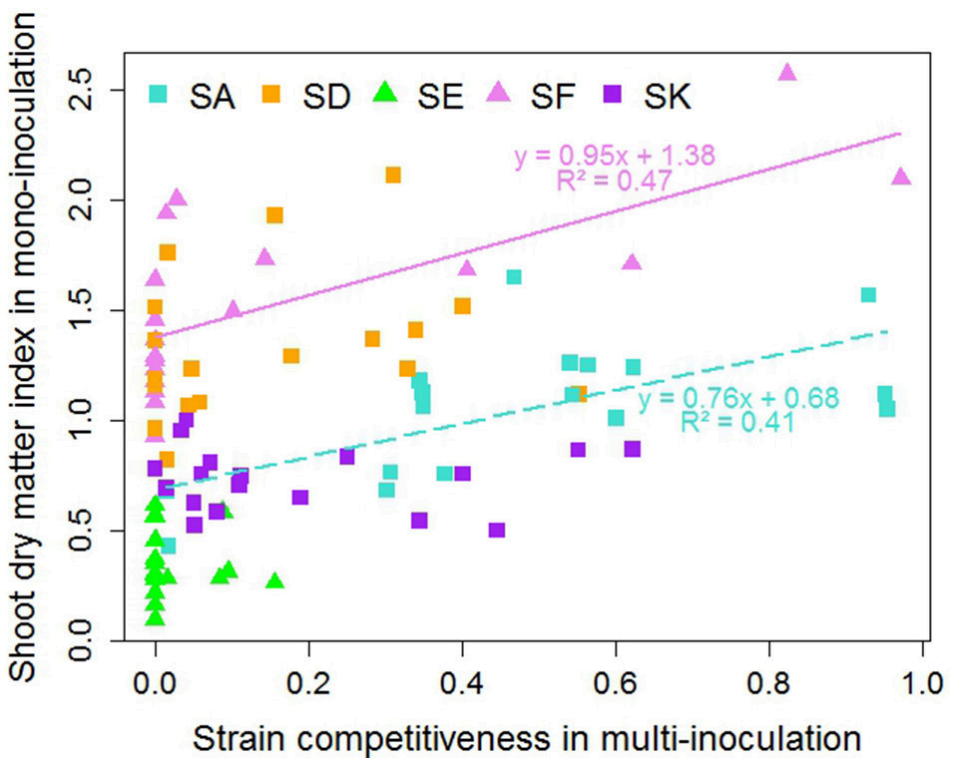
Relationship between shoot dry matter index in mono-inoculation (E2 experiment) and strain competitiveness for nodulation in multi-inoculation (E1 experiment) for 18 pea accessions. Squares represent European strains. Lines and equations represent significant linear regression results (both *P* < 0.05).

Overall differences in shoot dry matter index were associated with the Rlv strain (Figure 6). Globally, SF and SD promoted the highest biomass production and SE was the least efficient in all cases. Some differences in efficiency were observed depending on the pea accession; SF being for instance the most efficient for two pea accessions (R069, R098) and SA for the two accessions belonging to K04 (R046, R043). However, competitiveness and shoot dry matter index were found to be poorly correlated (Figure 6, Table S9). Significant but dispersed correlations were observed only for the two strains SF (*r*^2^ = 0.47, *p* = 0.001) and SA (*r*^2^ = 0.41, *p* = 0.002). For both strains, the slope of the regression line was lower than one indicating that increase in shoot dry matter index was associated with moderate change in competitiveness.

## Discussion

To our knowledge, this is the first time that the genetic diversity for pea-Rlv partner choice has been investigated within a pea collection representative of the variability within the genus *Pisum* and co-inoculated with a mixture of diverse Rlv strains. Little is known about the mechanisms underlying the competitiveness of Rlv strains for pea nodulation and the selection effected by pea genotypes faced with diverse compatible Rlv strains. Whether the competitiveness for nodulation of pea-Rlv symbiotic association is related to nitrogen fixation efficiency has been so far under-investigated.

### Nodulation is strongly correlated to shoot growth

Some differences in shoot dry matter were observed between the pea accessions according to their cultivation status or end-use, thus giving an overview of the modifications which have occurred during domestication and subsequent breeding history. Consistently, the landraces of *P. sativum* or *P. s*. subsp. *abyssinicum* form larger plants than the *P. fulvum* accessions and most of the other wild peas, and the low biomasses observed in dry peas are consistent with the breeding evolution toward field production of seeds for animal feeding. However, significant positive correlations between shoot biomass and nodule biomass or number were observed independently of species affiliation and breeding history. A close correlation between symbiotic organ development and plant growth has already been observed using samples exhibiting less genetic diversity within pea or *M. truncatula* (Bourion et al., 2010; Voisin et al., 2010; Laguerre et al., 2012). Pea and *M. truncatula* form indeterminate nodules, following two sequential processes: nodule formation and mature nodule expansion. Nodule formation results from early plant x rhizobia interaction. It occurs under situations of whole plant N limitation and is repressed by high N supply (Jeudy et al., 2010; Voisin et al., 2010). Nodule expansion is also strongly dependent on a systemic N-signaling regulation (Laguerre et al., 2012). In our study (moderate nitrogen supply) shoot biomass was more closely adjusted to nodule biomass than to nodule number. This suggests that pea adjusts its symbiotic capacity to its N demand by modulating C allocation for the expansion of nodules. Nodule biomass is therefore more linked to late adjustment processes than to nodule number.

### Partner choice varies according to genetic diversity

The “wild peas” group presented more variation with regard to symbiotic partner choice than cultivated peas, in agreement with previous observations (Lie, 1978; Lie et al., 1987). Within the “wild” D1 group, the highest diversity in partner choice was observed in the K01 cluster comprising the most primitive peas (i.e., *P. fulvum* accessions and peas representative of the early domestication events such as *P. sativum* subsp. *abyssinicum* and *P. sativum* accessions of the Middle East genetic diversity center). The K02 cluster was representative of peas from Afghanistan and India, and comprised the landrace peas resistant to nodulation with the European strains SA, SD, and SK, and highly specific to nodulation with SF/TOM. All the accessions of K03 originate from Abyssinia or Lybia, and display a high preference for the SA strain. This tight association between a European strain and African peas has not been previously reported. All these observations suggest that the domestication process and subsequent spread of peas from the Fertile Crescent may have resulted in a loss of the ability to establish symbiosis with some rhizobial strains. The cultivated pea groups (D2 and D3) encompass a great variety of end-uses, sowing types (spring vs. winter) and represent various steps in breeding history. Yet, no differences in partner choice are apparent among them: they essentially all choose predominantly SA. This does not support the idea that breeding practices under increasingly N-rich soils have led to a change in choice patterns between old and recent cultivars in pea, as seen in soybean (Kiers et al., 2007).

### The nodulation ability of a strain does not predict its competitiveness for nodulation

Experiments of mono- and multi-inoculation give complementary information and new insights into the natural variability of pea-Rlv partner choice. Our mono-inoculation results indicated that SF (TOM) is required for the nodulation of *P. humile* JI241 (R069) from Israel and for some Afghan pea accessions (R098, R029), confirming previously reported cases of host-controlled nodulation restriction (Lie, 1978; Young and Matthews, 1982). We also found in agreement with these authors that some other Afghan peas are only partially resistant or even susceptible to European Rlv strains. We even found further evidence for partially resistant accessions in neighboring countries (in the Caucasus). We confirmed the ability of SF/TOM, in a single-inoculum environment, to nodulate a wide spectrum of pea accessions (Lie, 1981; Young et al., 1982). However, we observed that SF, when inoculated in a mixture with the other four Rlv strains, was competitive only with the resistant accessions but not with the susceptible ones. The inhibiting effect of European strains on nodulation by SF/TOM, named competitive nodulation blocking (cnb), has been well documented on the resistant pea line “cv. Afghanistan” (Winarno and Lie, 1979; Dowling et al., 1989; Hogg et al., 2002). Evidence was provided that the high levels of Nod factors produced by these cnb+ European strains account for their nodulation blocking of “cv. Afghanistan” (Hogg et al., 2002). Our observation of the inhibition by four Rlv strains of nodulation by SF of susceptible peas does indicate that another competitiveness mechanism exists besides the cnb+ effect. Moreover, no correlation was observed for the four strains other than SF/TOM between their nodulation ability and their competitiveness for nodulation when inoculated in a mixture. All these results highlight that nodulation competitiveness is controlled by multiple genetic factors from both pea and rhizobia.

Other LysM-RLKs pea genes besides *SYM2* have been shown to be required for Nod factor perception (Madsen et al., 2003; Zhukov et al., 2008). Whether the variation in Nod factor perception together with the strain-dependent variation in Nod factor production is responsible for changes in competitiveness of various compatible pea-Rlv associations merits investigation. Furthermore, little is known about the role in pea-Rlv symbiosis of the control mechanisms revealed in model legumes and involving either rhizobial cell surface polysaccharides or secretion of rhizobial effector proteins (Masson-Boivin et al., 2009; Downie, 2010; Kawaharada et al., 2015; Malkov et al., 2016).

### Competitiveness for nodulation and nitrogen fixation efficiency are not correlated

It is conceivable that competitiveness for nodulation and efficiency of symbiotic association have been co-selected, favoring the best growing symbiotic plants. However, we found very little evidence that competitiveness is related to nitrogen fixation efficiency. A weak (albeit significant) relationship between strain competitiveness (multi-inoculation) and strain efficiency (mono-inoculated plants) was only found for two strains, SA and SF. This might arise mainly from differences between accessions in their level of resistance to nodulation by European strains and their specificity for SF. Thus, a high competitiveness of a given strain does not ensure high nitrogen-fixing efficiency and high biomass production for the plant. The several studies investigating the relationship between competitiveness and efficiency of symbiotic association have provided contrasted results according to the stage of observation. The poor relationship we observed is in agreement with the observation that Fix^−^ mutants of *R. meliloti* do not significantly differ in nodulation competitiveness with alfalfa (estimated 10 days after inoculation) from their Fix^+^ parental strains (Amarger, 1981). More recently we showed in *M. truncatula*/*Sinorhizobium* that plant N status does not impact initial partner choice for nodule formation but results in preferential later expansion of the nodules formed with the most efficient strains (Laguerre et al., 2012). Accordingly, a long-term preference for efficient strains of indigenous rhizobia was observed in 2 months-old alfalfa plants selected for high levels of nitrogen fixation (Hardarson et al., 1982). Preference for efficient associations requires late symbiotic interactions as a function of plant N demand, resulting in a strong allocation of metabolites by the plant to the nitrogen-fixing bacteroids. Conversely, competitiveness for nodulation is most probably determined during early plant-bacterial interactions and/or the bacterial colonization of the symbiotic organ but may not be directly related to the amount of N supplied through symbiosis. Our study highlighted that competitiveness for nodulation and nitrogen fixation efficiency must both be considered as selection criteria for improving pea crop production.

## Conclusion

Our work underlines the importance of viewing legumes as being exposed to several compatible symbiotic rhizobia rather than to a single strain. Mono-inoculation experiments are useful for evaluating the ability of a strain to form efficient symbiosis with a given host, but they fail to determine the ability of this strain to compete with other strains in a mixture, which is the situation commonly encountered in the field. A successful inoculant must not only provide enhanced symbiotic nitrogen fixation but be competitive for nodulation in the presence of a large population of indigenous rhizobia. Nodulation competitiveness is controlled by genetic factors from both the plant and the bacteria. Further experiments are in progress to decipher the genetic basis of this trait. Genome Wide Association Studies, involving larger pea and Rlv collections, increased genomic resources and higher throughput phenotyping technologies than those used in this study, may fulfill this challenge. Such knowledge of the key genetic factors involved will be essential for developing new pea varieties and Rlv inoculants for improved pea-Rlv symbiosis.

## Author contributions

VB and ML co-coordinated the overall study that was also initiated by Gisèle Laguerre. GD contributed to the conception of the study and the design of the pea collection. JB contributed to the conception of the study and coordinated the genotyping of the pea collection. MS performed pea collection genetic structure analyses. VB contributed to the design of the pea collection, coordinated and participated in greenhouse experiments and plant phenotypic data acquisition, performed plant phenotypic data analyses and interpretation. VA and KH-G participated in greenhouse experiments and contributed to phenotypic plant data acquisition. MC-M and CD provided seeds and information on the pea collection, and contributed to phenotypic data acquisition. BB and MP performed phylogenic analysis of the Rlv bacteria. BB, KH-G, ML, and PT collected nodules and performed analyses of bacterial occupancy. DV contributed to statistical analysis of the data. VB and ML wrote the manuscript which was revised and accepted by all authors.

### Conflict of interest statement

The authors declare that the research was conducted in the absence of any commercial or financial relationships that could be construed as a potential conflict of interest.

## References

[B1] Amarger N. (1981). Competition for nodule formation between effective and ineffective strains of *Rhizobium meliloti*. Soil Biol. Biochem. 13, 475–480. 10.1016/0038-0717(81)90037-7

[B2] Bourion V. Laguerre G. Depret G. Voisin A. S. Salon C. Duc G. (2007). Genetic variability in nodulation and root growth affects nitrogen fixation and accumulation in pea. Ann. Bot. 100, 589–598. 10.1093/aob/mcm147 17670753 PMC2533614

[B3] Bourion V. Rizvi S. M. H. Fournier S. de Larambergue H. Galmiche F. Marget P.. (2010). Genetic dissection of nitrogen nutrition in pea through a QTL approach of root, nodule, and shoot variability. Theor. Appl. Genet. 121, 71–86. 10.1007/s00122-010-1292-y 20180092

[B4] Bremer E. Rennie R. J. Rennie D. A. (1988). Dinitrogen fixation of lentil, field pea and fababean under drylands conditions. Can. J. Soil Sci. 68, 553–562. 10.4141/cjss88-053

[B5] Brewin N. Beringer J. Johnston A. (1980). Plasmid-mediated transfer of host-range specificity between two strains of *Rhizobium leguminosarum*. Microbiology 120, 413–420. 10.1099/00221287-120-2-413

[B6] Brewin N. J. Wood E. A. Young J. P. W. (1983). Contribution of the symbiotic plasmid to the competitiveness of *Rhizobium leguminosarum*. J. Gen. Microbiol. 129, 2973–2977. 10.1099/00221287-129-10-2973

[B7] Burstin J. Salloignon P. Chabert-Martinello M. Magnin-Robert J. B. Siol M. Jacquin F.. (2015). Genetic diversity and trait genomic prediction in a pea diversity panel. BMC Genomics 16:105. 10.1186/s12864-015-1266-1 25765216 PMC4355348

[B8] Cousin R. (1997). Peas (*Pisum sativum* L.). Field Crops Res. 53, 111–130. 10.1016/S0378-4290(97)00026-9

[B9] Davis E. O. Evans I. J. Johnston A. W. B. (1988). Identification of *nodX*, a gene that allows *Rhizohium leguminosarum* biovar *viciae* strain TOM to nodulate Afghanistan peas. Mol. Gen. Genet. 212, 531–535. 10.1007/BF00330860 3419422

[B10] Dénarié J. Debellé F. Rosenberg C. (1992). Signaling and host range variation in nodulation. Annu. Rev. Microbiol. 46, 497–531. 10.1146/annurev.mi.46.100192.002433 1444265

[B11] D'Haeze W. Holsters M. (2002). Nod factor structures, responses, and perception during initiation of nodule development. Glycobiology 12, 79R−105R. 10.1093/glycob/12.6.79R 12107077

[B12] Dowling D. N. Stanley J. Broughton W. J. (1989). Competitive nodulation blocking of Afghanistan pea is determined by *nodDABC* and *nodFE* alleles in *Rhizobium leguminosarum*. Mol. Gen. Genet. 216, 170–174. 10.1007/bf00332247

[B13] Downie J. A. (2010). The roles of extracellular proteins, polysaccharides and signals in the interactions of rhizobia with legume roots. FEMS Microbiol. Rev. 34, 150–170. 10.1111/j.1574-6976.2009.00205.x 20070373

[B14] Duc G. Agrama H. Bao S. Y. Berger J. Bourion V. De Ron A. M. (2015). Breeding annual grain legumes for sustainable agriculture: new methods to approach complex traits and target new cultivar ideotypes. CRC. Crit. Rev. Plant Sci. 34, 381–411. 10.1080/07352689.2014.898469

[B15] Fesenko A. N. Provorov N. A. Orlova I. F. Orlov V. P. Simarov B. V. (1995). Selection of *Rhizobium leguminosarum* bv. viceae strains for inoculation of *Pisum sativum* L. cultivars - analysis of symbiotic efficiency and nodulation competitiveness. Plant Soil 172, 189–198. 10.1007/BF00011321

[B16] Fujita H. Aoki S. Kawaguchi M. (2014). Evolutionary dynamics of nitrogen fixation in the legume–rhizobia symbiosis. PLoS ONE 9:e93670. 10.1371/journal.pone.0093670 24691447 PMC3972148

[B17] Galloway J. N. Townsend A. R. Erisman J. W. Bekunda M. Cai Z. Freney J. R.. (2008). Transformation of the Nitrogen cycle: recent trends, questions, and potential solutions. Science 320, 889–892. 10.1126/science.1136674 18487183

[B18] Hardarson G. Heichel G. H. Barnes D. K. Vance C. P. (1982). Rhizobial strain preference of alfalfa populations selected for characteristics associated with N_2_ fixation. Crop Sci. 22, 55–58. 10.2135/cropsci1982.0011183X002200010012x

[B19] Heath K. D. Tiffin P. (2009). Stabilizing mechanisms in a legume–rhizobium mutualism. Evolution 63, 652–662. 10.1111/j.1558-5646.2008.00582.x 19087187

[B20] Herridge D. F. Peoples M. B. Boddey R. M. (2008). Global inputs of biological nitrogen fixation in agricultural systems. Plant Soil 311, 1–18. 10.1007/s11104-008-9668-3

[B21] Hogg B. Davies A. E. Wilson K. E. Bisseling T. Downie J. A. (2002). Competitive nodulation blocking of cv. Afghanistan pea is related to high levels of nodulation factors made by some strains of *Rhizobium leguminosarum* bv. *viciae*. Mol. Plant Microbe Interact. 15, 60–68. 10.1094/mpmi.2002.15.1.60 11843305

[B22] Holl F. B. (1975). Host plant control of the inheritance of dinitrogen fixation in the *Pisum*-*Rhizobium* symbiosis. Euphytica 24, 767–770. 10.1007/BF00132916

[B23] Jakobsson M. Rosenberg N. A. (2007). CLUMPP: a cluster matching and permutation program for dealing with label switching and multimodality in analysis of population structure. Bioinformatics 23, 1801–1806. 10.1093/bioinformatics/btm233 17485429

[B24] Jensen E. S. Hauggaard-Nielsen H. (2003). How can increased use of biological N_2_ fixation in agriculture benefit the environment? Plant Soil 252, 177–186. 10.1023/A:1024189029226

[B25] Jeudy C. Ruffel S. Freixes S. Tillard P. Santoni A. L. Morel S.. (2010). Adaptation of *Medicago truncatula* to nitrogen limitation is modulated via local and systemic nodule developmental responses. New Phytologist 185, 817–828. 10.1111/j.1469-8137.2009.03103.x 20015066

[B26] Jing R. Vershinin A. Grzebyta J. Shaw P. Smýkal P. Marshall D.. (2010). The genetic diversity and evolution of field pea (*Pisum*) studied by high throughput retrotransposon based insertion polymorphism (RBIP) marker analysis. BMC Evol. Biol. 10:44. 10.1186/1471-2148-10-44 20156342 PMC2834689

[B27] Jombart T. Devillard S. Balloux F. (2010). Discriminant analysis of principal components: a new method for the analysis of genetically structured populations. BMC Genet. 11:94. 10.1186/1471-2156-11-94 20950446 PMC2973851

[B28] Kawaharada Y. Kelly S. Nielsen M. W. Hjuler C. T. Gysel K. Muszynski A.. (2015). Receptor-mediated exopolysaccharide perception controls bacterial infection. Nature 523, 308–315. 10.1038/nature14611 26153863

[B29] Kiers E. T. Hutton M. G. Denison R. F. (2007). Human selection and the relaxation of legume defences against ineffective rhizobia. Proc. R. Soc. B Biol. Sci. 274, 3119–3126. 10.1098/rspb.2007.1187 17939985 PMC2293947

[B30] Kumar N. Lad G. Giuntini E. Kaye M. E. Udomwong P. Shamsani N. J. (2015). Bacterial genospecies that are not ecologically coherent: population genomics of *Rhizobium leguminosarum*. Open Biol. 5:140133 10.1098/rsob.140133 25589577 PMC4313370

[B31] Laguerre G. Depret G. Bourion V. Duc G. (2007). *Rhizobium leguminosarum* bv. viciae genotypes interact with pea plants in developmental responses of nodules, roots and shoots. New Phytol. 176, 680–690. 10.1111/j.1469-8137.2007.02212.x 17822397

[B32] Laguerre G. Heulin-Gotty K. Brunel B. Klonowska A. Le Quéré A. Tillard P.. (2012). Local and systemic N signaling are involved in *Medicago truncatula* preference for the most efficient *Sinorhizobium* symbiotic partners. New Phytol. 195, 437–449. 10.1111/j.1469-8137.2012.04159.x 22548481

[B33] Laguerre G. Louvrier P. Allard M. R. Amarger N. (2003). Compatibility of rhizobial genotypes within natural populations of *Rhizobium leguminosarum* biovar viciae for nodulation of host legumes. Appl. Environ. Microbiol. 69, 2276–2283. 10.1128/AEM.69.4.2276-2283.2003 12676710 PMC154822

[B34] Laguerre G. Nour S. M. Macheret V. Sanjuan J. Drouin P. Amarger N. (2001). Classification of rhizobia based on *nodC* and *nifH* gene analysis reveals a close phylogenetic relationship among *Phaseolus vulgaris* symbionts. Microbiology 147, 981–993. 10.1099/00221287-147-4-981 11283294

[B35] Liang Y. Tóth K. Cao Y. R. Tanaka K. Espinoza C. Stacey G. (2014). Lipochitooligosaccharide recognition: an ancient story. New Phytol. 204, 289–296. 10.1111/nph.12898 25453133

[B36] Lie T. A. (1978). Symbiotic specialisation in pea plants: the requirement of specific *Rhizobium* strains for peas from Afghanistan. Ann. Appl. Biol. 88, 462–465. 10.1111/j.1744-7348.1978.tb00743.x

[B37] Lie T. A. (1981). Gene centres, a source for genetic variants in symbiotic nitrogen fixation: host-induced ineffectivity in *Pisum sativum* ecotype fulvum. Plant Soil 61, 125–134. 10.1007/BF02277369

[B38] Lie T. A. Goktan D. Engin M. Pijnenborg J. Anlarsal E. (1987). Coevolution of the legume-Rhizobium association. Plant. Soil. 100, 171–181. 10.1007/BF02370940

[B39] Madsen E. B. Madsen L. H. Radutoiu S. Olbryt M. Rakwalska M. Szczyglowski K.. (2003). A receptor kinase gene of the LysM type is involved in legume perception of rhizobial signals. Nature 425, 637–640. 10.1038/nature02045 14534591

[B40] Malkov N. Fliegmann J. Rosenberg C. Gasciolli V. Timmers A. C. Nurisso A.. (2016). Molecular basis of lipo-chitooligosaccharide recognition by the lysin motif receptor-like kinase LYR3 in legumes. Biochem. J. 473, 1369–1378. 10.1042/BCJ20160073 26987814

[B41] Marco D. E. Carbajal J. P. Cannas S. Pérez-Arnedo R. Hidalgo-Perea Á. Olivares J.. (2009). An experimental and modelling exploration of the host-sanction hypothesis in legume–rhizobia mutualism. J. Theor. Biol. 259, 423–433. 10.1016/j.jtbi.2009.03.033 19358857

[B42] Masson-Boivin C. Giraud E. Perret X. Batut J. (2009). Establishing nitrogen-fixing symbiosis with legumes: how many rhizobium recipes? Trends Microbiol. 17, 458–466. 10.1016/j.tim.2009.07.004 19766492

[B43] McKenzie R. H. Middleton A. B. Solberg E. D. DeMulder J. Flore N. Clayton G. W. (2001). Response of pea to rhizobia inoculation and starter nitrogen in Alberta. Can. J. Plant Sci. 81, 637–643. 10.4141/P01-006

[B44] Meade J. Higgins P. Ogara F. (1985). Studies on the inoculation and competitiveness of a *Rhizobium leguminosarum* strain in soils containing indigenous rhizobia. Appl. Environ. Microbiol. 49, 899–903. 16346769 10.1128/aem.49.4.899-903.1985 PMC238466

[B45] Moreau D. Voisin A. S. Salon C. Munier-Jolain N. (2008). The model symbiotic association between *Medicago truncatula* cv. Jemalong and *Rhizobium meliloti* strain 2011 leads to N-stressed plants when symbiotic N_2_ fixation is the main N source for plant growth. J. Exp. Bot. 59, 3509–3522. 10.1093/jxb/ern203 18703494

[B46] Mutch L. A. Young J. P. W. (2004). Diversity and specificity of *Rhizobium leguminosarum* biovar *viciae* on wild and cultivated legumes. Mol. Ecol. 13, 2435–2444. 10.1111/j.1365-294X.2004.02259.x 15245415

[B47] Oono R. Anderson C. G. Denison R. F. (2011). Failure to fix nitrogen by non-reproductive symbiotic rhizobia triggers host sanctions that reduce fitness of their reproductive clonemates. Proc. R. Soc. B Biol. Sci. 278, 2698–2703. 10.1098/rspb.2010.2193 21270038 PMC3136820

[B48] Peix A. Ramirez-Bahena M. H. Velazquez E. Bedmar E. J. (2015). Bacterial associations with legumes. Crit. Rev. Plant Sci. 34, 17–42. 10.1080/07352689.2014.897899

[B49] Phillips D. A. (1980). Efficiency of symbiotic nitrogen fixation in legumes. Annu. Rev. Plant Physiol. 31, 29–49. 10.1146/annurev.pp.31.060180.000333 PMC542628 16660105

[B50] Raj A. Stephens M. Pritchard J. K. (2014). fastSTRUCTURE: variational inference of population structure in large SNP data sets. Genetics 197, U573–U207. 10.1534/genetics.114.164350 24700103 PMC4063916

[B51] Sachs J. L. Ehinger M. O. Simms E. L. (2010). Origins of cheating and loss of symbiosis in wild *Bradyrhizobium*. J. Evol. Biol. 23, 1075–1089. 10.1111/j.1420-9101.2010.01980.x 20345811

[B52] Sachs J. L. Kembel S. W. Lau A. H. Simms E. L. (2009). *In Situ* phylogenetic structure and diversity of wild *Bradyrhizobium* communities. Appl. Environ. Microbiol. 75, 4727–4735. 10.1128/AEM.00667-09 19482951 PMC2708436

[B53] Schulze J. Adgo E. Merbach W. (1999). Carbon costs associated with N_2_ fixation in *Vicia faba* L and *Pisum sativum* L. over a 14-day period. Plant Biol. 1, 625–631. 10.1111/j.1438-8677.1999.tb00273.x

[B54] Simms E. L. Taylor D. L. (2002). Partner choice in nitrogen-fixation mutualisms of legumes and rhizobia. Integr. Comp. Biol. 42, 369–380. 10.1093/icb/42.2.369 21708730

[B55] Smykal P. Coyne C. J. Ambrose M. J. Maxted N. Schaefer H. Blair M. W. (2015). Legume crops phylogeny and genetic diversity for science and breeding. Crit. Rev. Plant Sci. 34, 43–104. 10.1080/07352689.2014.897904

[B56] Smykal P. Kenicer G. Flavell A. J. Corander J. Kosterin O. Redden R. J. (2011). Phylogeny, phylogeography and genetic diversity of the *Pisum* genus. Plant Genet. Resour. 9, 4–18. 10.1017/S147926211000033X

[B57] Spaink H. P. Sheeley D. M. Vanbrussel A. A. N. Glushka J. York W. S. Tak T.. (1991). A novel highly unsaturated fatty-acid moiety of lipo-oligosaccharide signals determines host specificity of *Rhizobium*. Nature 354, 125–130. 10.1038/354125a0 1944592

[B58] Tayeh N. Aluome C. Falque M. Jacquin F. Klein A. Chauveau A.. (2015). Development of two major resources for pea genomics: the GenoPea 13.2K SNP Array and a high-density, high-resolution consensus genetic map. Plant J. 84, 1257–1273. 10.1111/tpj.13070 26590015

[B59] Trainer M. A. Charles T. C. (2006). The role of PHB metabolism in the symbiosis of rhizobia with legumes. Appl. Microbiol. Biotechnol. 71, 377–386. 10.1007/s00253-006-0354-1 16703322

[B60] Triplett E. W. Sadowsky M. J. (1992). Genetics of competition for nodulation of legumes. Annu. Rev. Microbiol. 46, 399–428. 10.1146/annurev.mi.46.100192.002151 1444262

[B61] Vershinin A. V. Allnutt T. R. Knox M. R. Ambrose M. J. Ellis T. H. N. (2003). Transposable elements reveal the impact of introgression, rather than transposition, in *Pisum* diversity, evolution, and domestication. Mol. Biol. Evol. 20, 2067–2075. 10.1093/molbev/msg220 12949152

[B62] Voisin A. S. Bourion V. Duc G. Salon C. (2007). Using an ecophysiological analysis to dissect genetic variability and to propose an ideotype for nitrogen nutrition in pea. Ann. Bot. 100, 1525–1536. 10.1093/aob/mcm241 17921490 PMC2759225

[B63] Voisin A. S. Munier-Jolain N. G. Salon C. (2010). The nodulation process is tightly adjusted to plant growth. An analysis using environmentally and genetically induced variation of nodule number and biomass in pea. Plant Soil 337, 399–412. 10.1007/s11104-010-0536-6

[B64] Walker S. A. Viprey V. Downie J. A. (2000). Dissection of nodulation signaling using pea mutants defective for calcium spiking induced by Nod factors and chitin oligomers. Proc. Natl. Acad. Sci. U.S.A. 97, 13413–13418. 10.1073/pnas.230440097 11078515 PMC27238

[B65] Winarno R. Lie T. A. (1979). Competition between Rhizobium strains in nodule formation: Interaction between nodulating and non-nodulating strains. Plant Soil 51, 135–142. 10.1007/BF02205933

[B66] Young J. P. W. Johnston A. W. B. Brewin N. J. (1982). A search for peas (*Pisum sativum* L.) showing strain specificity for symbiotic *Rhizobium leguminosarum*. Heredity 48, 197–201. 10.1038/hdy.1982.25

[B67] Young J. P. W. Matthews P. (1982). A distinct class of peas (*Pisum sativum* L.) from Afghanistan that show strain specificity for symbiotic Rhizobium. Heredity 48, 203–210. 10.1038/hdy.1982.26

[B68] Zhukov V. Radutoiu S. Madsen L. H. Rychagova T. Ovchinnikova E. Borisov A.. (2008). The pea *Sym37* receptor kinase gene controls infection-thread initiation and nodule development. Mol. Plant 21, 1600–1608. 10.1094/MPMI-21-12-1600 18986256

[B69] Zohary D. Hopf M. (1973). Domestication of pulses in the old world. Science 182, 887–894. 10.1126/science.182.4115.887 17737521

