# Supplementary material for: Co-inoculation of a Pea Core-Collection with Diverse Rhizobial Strains Shows Competitiveness for Nodulation and Efficiency of Nitrogen Fixation Are Distinct traits in the Interaction
Source: Front Plant Sci. 2018 Jan 10;8:2249. doi: 10.3389/fpls.2017.02249 (PMC5767787; doi:10.3389/fpls.2017.02249)
Supplement: Supplementary file 10 [file Image1.pdf]

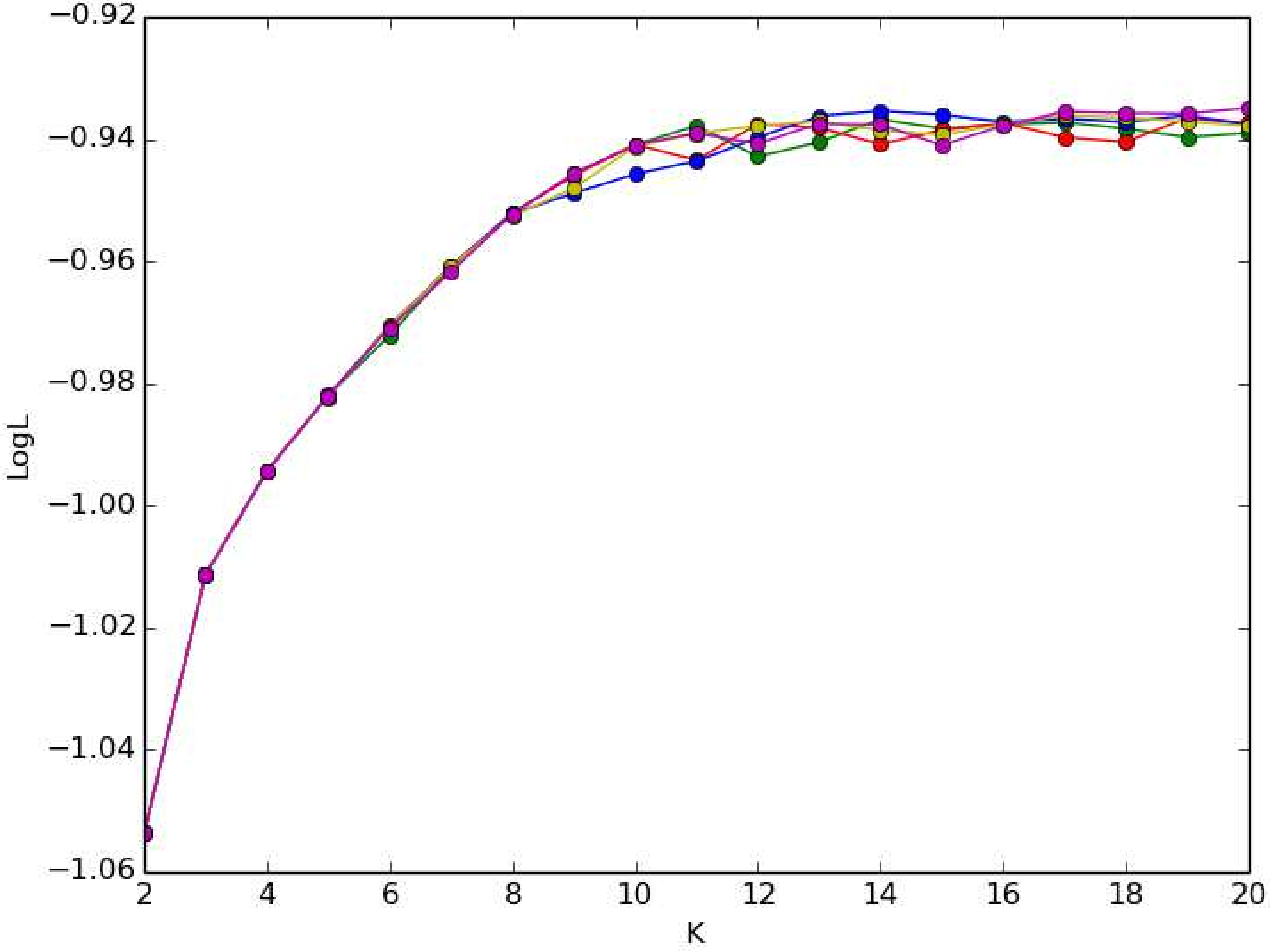

Figure S1: fastSTRUCTURE analysis. Log Likelihood of the model as a function of the number of clusters for each of the five runs
