# Supplementary figures and images for: Co-inoculation of a Pea Core-Collection with Diverse Rhizobial Strains Shows Competitiveness for Nodulation and Efficiency of Nitrogen Fixation Are Distinct traits in the Interaction
Source: Front Plant Sci. 2018 Jan 10;8:2249. doi: 10.3389/fpls.2017.02249 (PMC5767787; doi:10.3389/fpls.2017.02249)

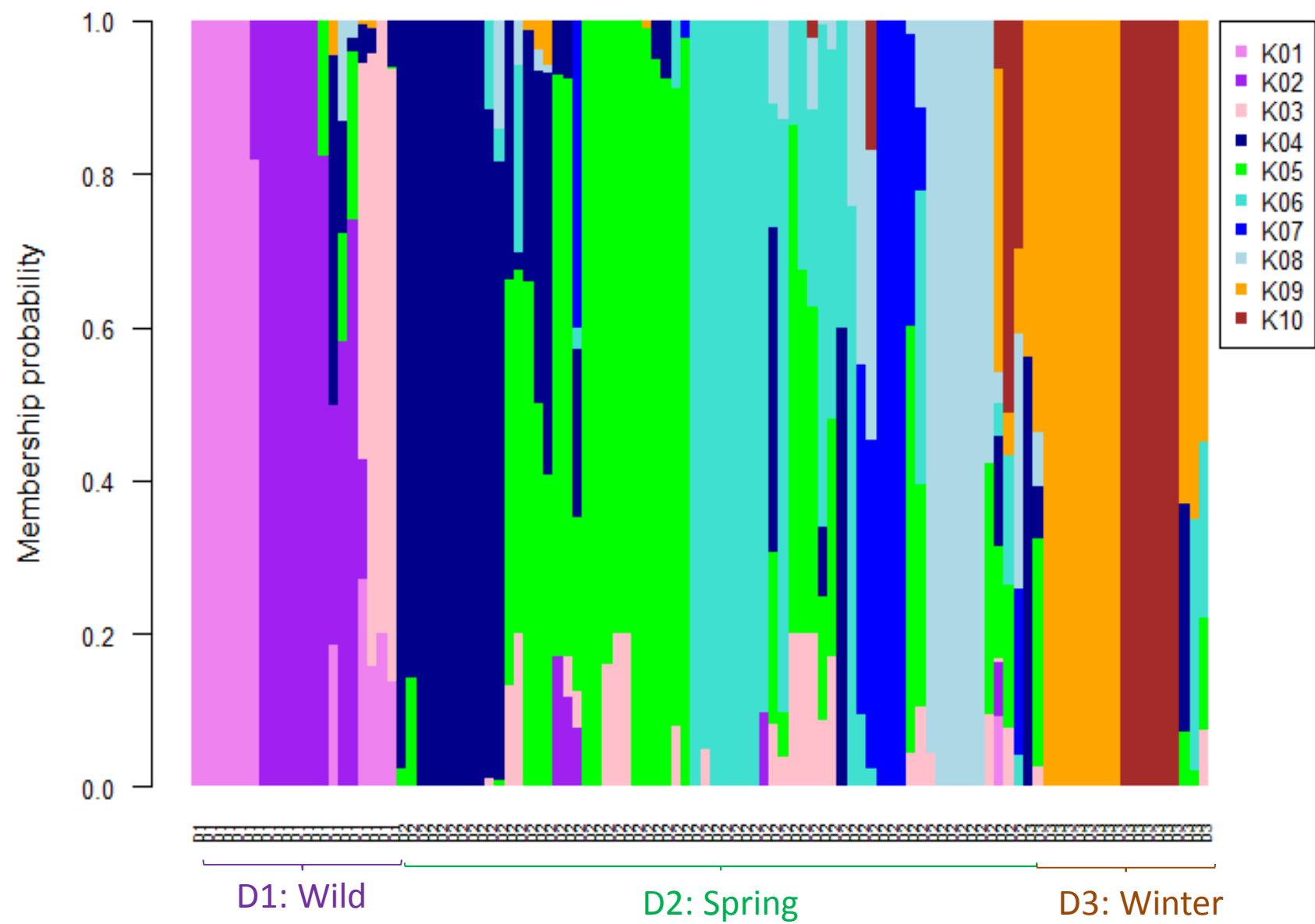

**Figure S2:** Genetic structure of the pea collection using the fastSTRUCTURE analysis

Supplement: Supplementary file 11 [file Image2.PDF]

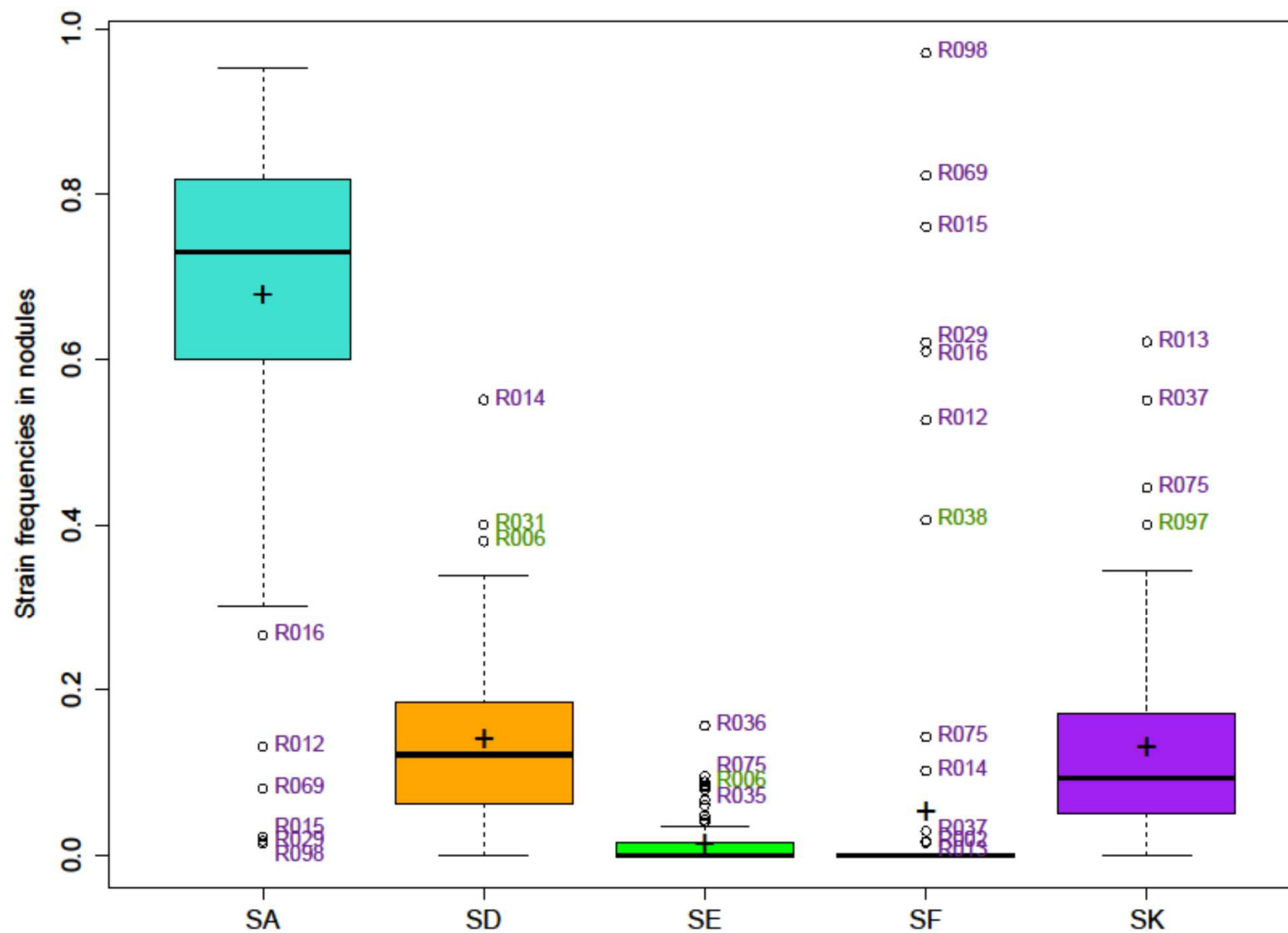

**Figure S5:** Strain frequencies in the nodules of 104 pea accessions (E1 experiment).

Supplement: Supplementary file 14 [file Image5.PDF]
