# Supplementary material for: Co-inoculation of a Pea Core-Collection with Diverse Rhizobial Strains Shows Competitiveness for Nodulation and Efficiency of Nitrogen Fixation Are Distinct traits in the Interaction
Source: Front Plant Sci. 2018 Jan 10;8:2249. doi: 10.3389/fpls.2017.02249 (PMC5767787; doi:10.3389/fpls.2017.02249)

Figure S3: Molecular Phylogenetic analysis of *nodD* sequences (651 nt) by Maximum Likelihood method among Rhizobium strains sv. viciae (total of 104 sequences). Genbank accession numbers are given in front the strain numbers. Bar represents 5% sequence divergence. These analyses were conducted using the MEGA7 software (Kumar et al., 2016).

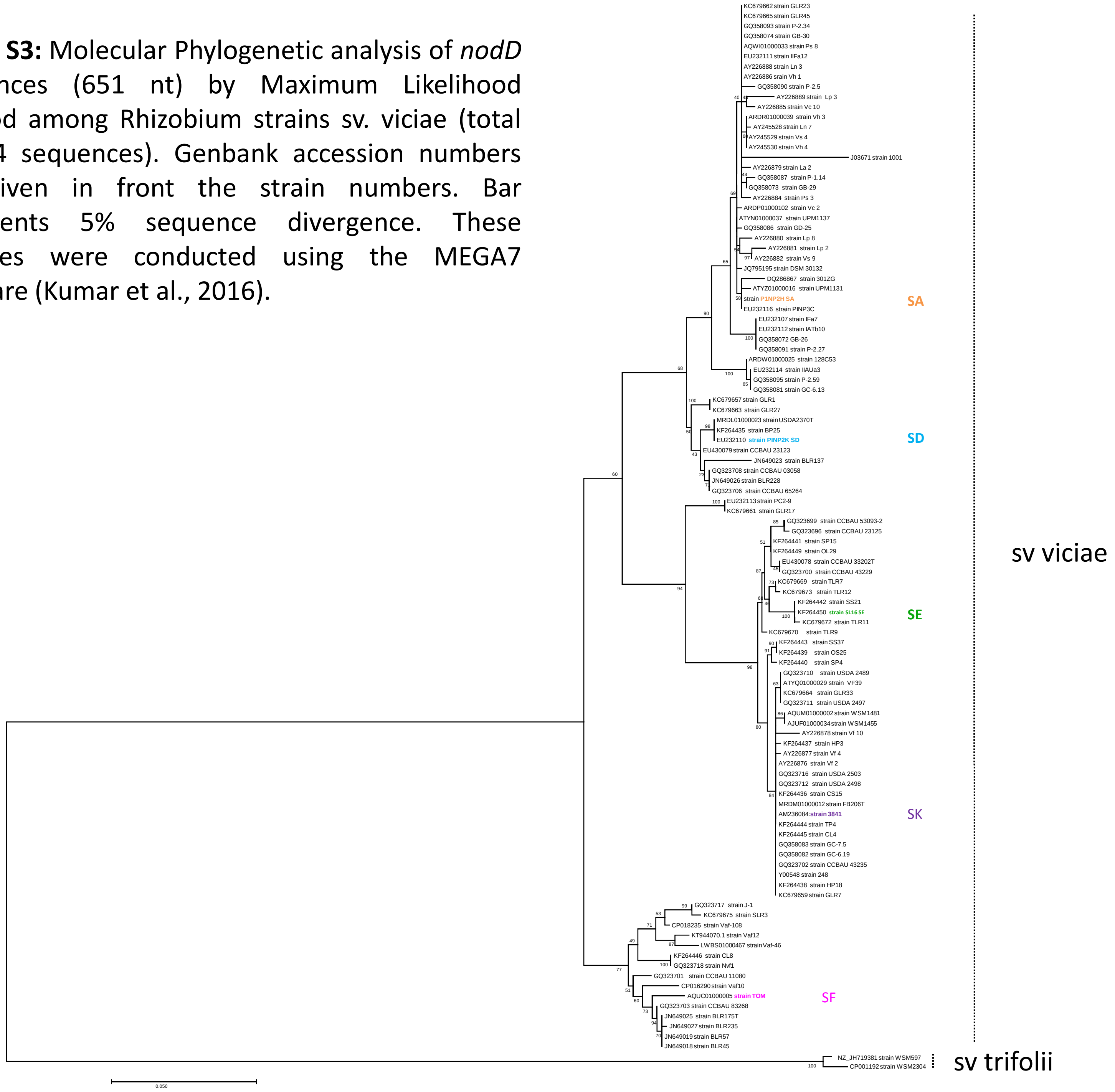

Supplement: Supplementary file 12 [file Image3.PDF]
