# Supplementary material for: Co-inoculation of a Pea Core-Collection with Diverse Rhizobial Strains Shows Competitiveness for Nodulation and Efficiency of Nitrogen Fixation Are Distinct traits in the Interaction
Source: Front Plant Sci. 2018 Jan 10;8:2249. doi: 10.3389/fpls.2017.02249 (PMC5767787; doi:10.3389/fpls.2017.02249)
Supplement: Supplementary file 13 [file Image4.PDF]

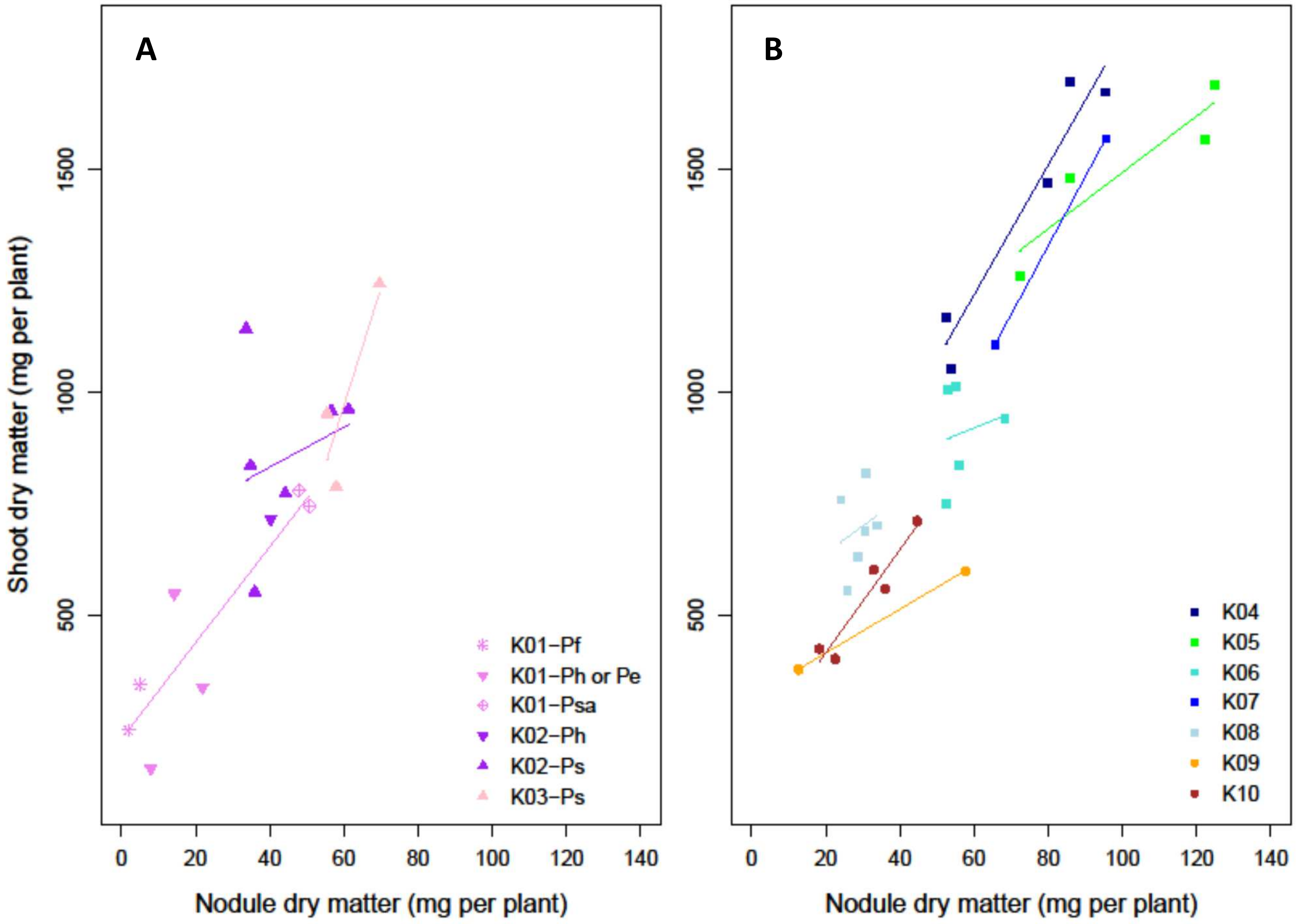

Figure S4: Relationship between shoot dry matter and nodule dry matter, (A) for the 17 pea accessions belonging to D1 (B) for the 29 pea cultivars belonging to D2 or D3, according to their membership to cluster groups in the multi-inoculation experiment (E1)
