# Supplementary material for: Co-inoculation of a Pea Core-Collection with Diverse Rhizobial Strains Shows Competitiveness for Nodulation and Efficiency of Nitrogen Fixation Are Distinct traits in the Interaction
Source: Front Plant Sci. 2018 Jan 10;8:2249. doi: 10.3389/fpls.2017.02249 (PMC5767787; doi:10.3389/fpls.2017.02249)
Supplement: Supplementary file 15 [file Image6.PDF]

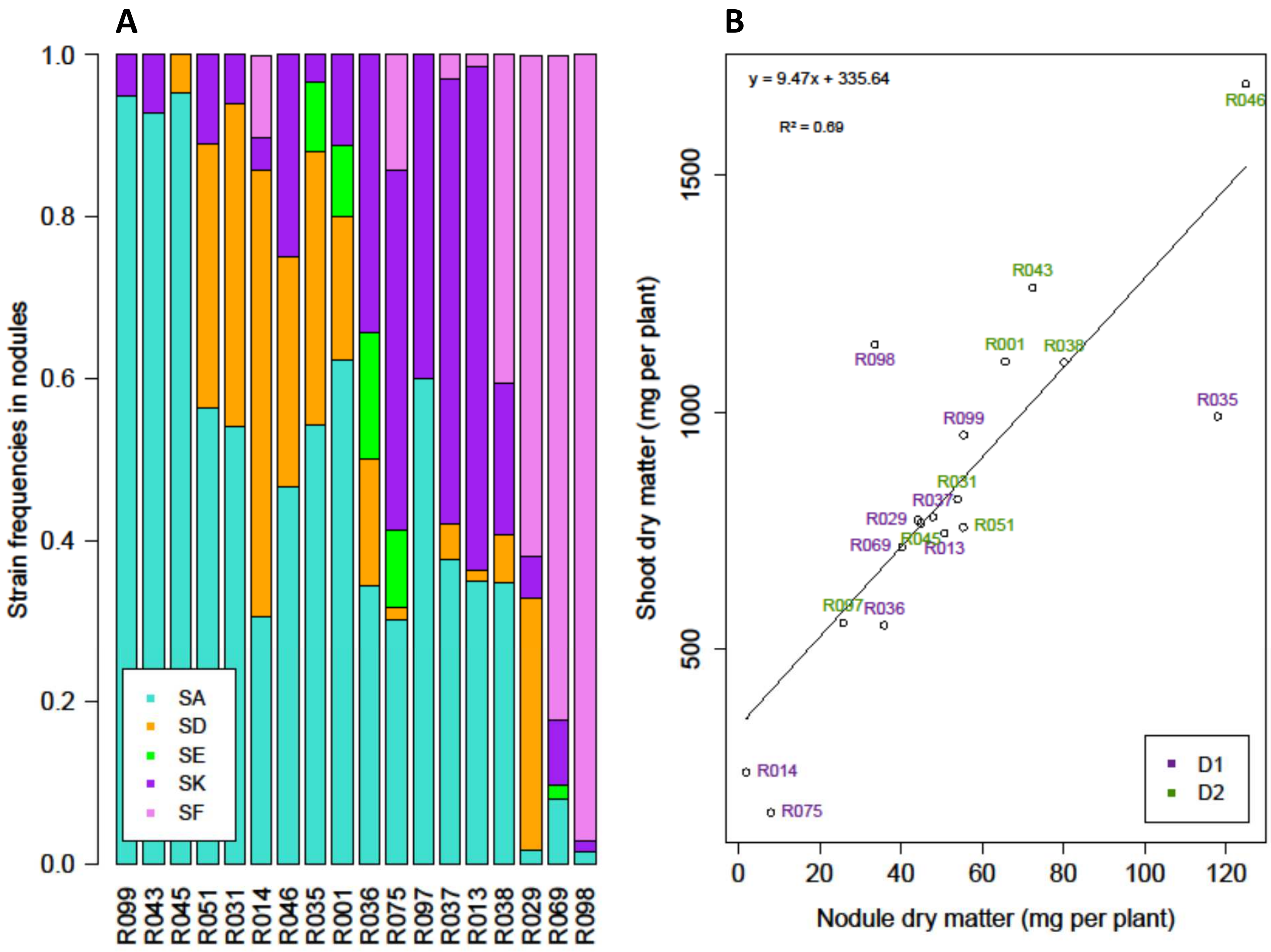

Figure S6: For 18 pea accessions multi-inoculated with a mixture of five Rlv strains (E1 experiment): (A) Strain frequencies in the nodules (B) Relationship between shoot dry matter and nodule dry matter
