# Supplementary material for: Co-inoculation of a Pea Core-Collection with Diverse Rhizobial Strains Shows Competitiveness for Nodulation and Efficiency of Nitrogen Fixation Are Distinct traits in the Interaction
Source: Front Plant Sci. 2018 Jan 10;8:2249. doi: 10.3389/fpls.2017.02249 (PMC5767787; doi:10.3389/fpls.2017.02249)
Supplement: Supplementary file 17 [file Image8.pdf]

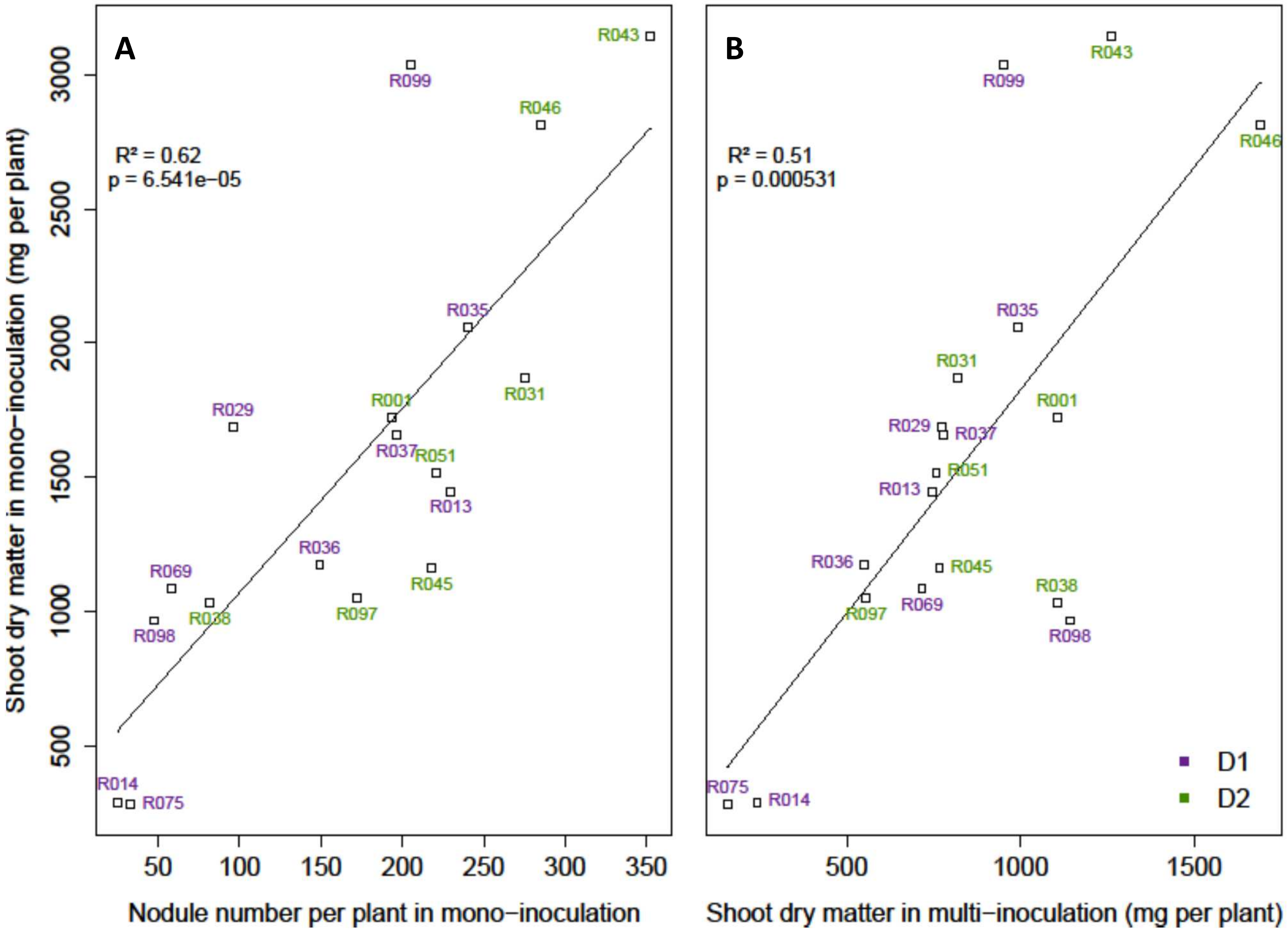

Figure S8: Relationship for 18 pea accessions between mean shoot dry matter in the mono-inoculation experiment (E2) and (A) mean nodule number in mono-inoculation experiment (E2) or (B) shoot dry matter in multi-inoculation experiment (E1).
